# Metformin ameliorates osteoporosis by enhancing bone angiogenesis via the YAP1/TAZ-HIF1α axis

**DOI:** 10.1186/s10020-025-01169-7

**Published:** 2025-03-30

**Authors:** Hao Yin, Zhe Ruan, Teng-Fei Wan, Zhi-Rou Lin, Chun-Yuan Chen, Zhen-Xing Wang, Jia Cao, Yi-Yi Wang, Ling Jin, Yi-Wei Liu, Guo-Qiang Zhu, Jiang-Shan Gong, Jing-Tao Zou, Yi Luo, Yin Hu, Zhao-Hui Li, Hao Luo, Yu-Qi Liu, Cheng Long, Shu-Shan Zhao, Yong Zhu, Hui Xie

**Affiliations:** 1 Department of Orthopedics, Xiangya Hospital, Central South University, Changsha, 410008 Hunan China; 2 Movement System Injury and Repair Research Center, Xiangya Hospital, Central South University, Changsha, 410008 Hunan China; 3 Department of Orthopedics, The First Hospital of Changsha, Changsha, 410008 Hunan China; 4 Department of Sports Medicine, Xiangya Hospital, Central South University, Changsha, 410008 Hunan China; 5 National Clinical Research Center for Geriatric Disorders, Xiangya Hospital, Central South University, Changsha, 410008 Hunan China; 6 Hunan Key Laboratory of Organ Injury, Aging and Regenerative Medicine, Changsha, 410008 Hunan China; 7 Hunan Key Laboratory of Bone Joint Degeneration and Injury, Changsha, 410008 Hunan China; 8 The First Affiliated Hospital, Department of Metabolism and Endocrinology, Hengyang Medical School, University of South China, Hengyang, 421001 Hunan China

**Keywords:** Metformin, Osteoporosis, YAP1/TAZ, HIF1α, Angiogenesis, Type H vessel

## Abstract

**Background:**

Osteoporosis, resulting from an imbalance between osteoclast-mediated bone resorption and osteoblast-mediated bone formation, affects millions globally. Recent studies have identified type H vessels (CD31^hi^EMCN^hi^) as a specialized subset of bone blood vessels that positively regulate bone formation. This study aims to investigate the effects of metformin on bone mass, strength, and angiogenesis in osteoporotic mice, and to elucidate the underlying molecular mechanisms, particularly focusing on the YAP1/TAZ-HIF1α axis.

**Methods:**

Osteoporotic mice were administered metformin, and bone mass and strength were measured. In vivo and in vitro angiogenesis assays were performed under hypoxic conditions. Expression levels of YAP1/TAZ and HIF1α were assessed in femoral metaphysis and hypoxia-cultured human microvascular endothelial cells (HMECs). Small interfering RNA was used to interfere with HIF1α or YAP1/TAZ expression in hypoxia-cultured HMECs. Additionally, we employed AAV-mediated overexpression of YAP1/TAZ in vivo to determine whether elevated YAP1/TAZ levels alter metformin’s effects on bone mass and angiogenesis.

**Results:**

Metformin significantly enhanced bone mass and strength in osteoporotic mice. It also promoted angiogenesis under hypoxia conditions both in vivo and in vitro. Metformin reduced YAP1/TAZ expression while increasing HIF1α expression in both the femoral metaphysis of osteoporotic mice and hypoxia-cultured HMECs. Interference with HIF1α or YAP1/TAZ confirmed that metformin enhances HIF1α and its target genes primarily by inhibiting YAP1/TAZ. Furthermore, overexpression of YAP1/TAZ partially reversed the bone-protective effect of metformin, leading to reduced HIF1α levels and diminished type H vessel formation.

**Conclusion:**

Our findings suggest that metformin holds promise as a therapeutic agent for osteoporosis by enhancing type H vessel formation through the inhibition of the YAP1/TAZ-HIF1α axis.

**Supplementary Information:**

The online version contains supplementary material available at 10.1186/s10020-025-01169-7.

## Graphical abstract

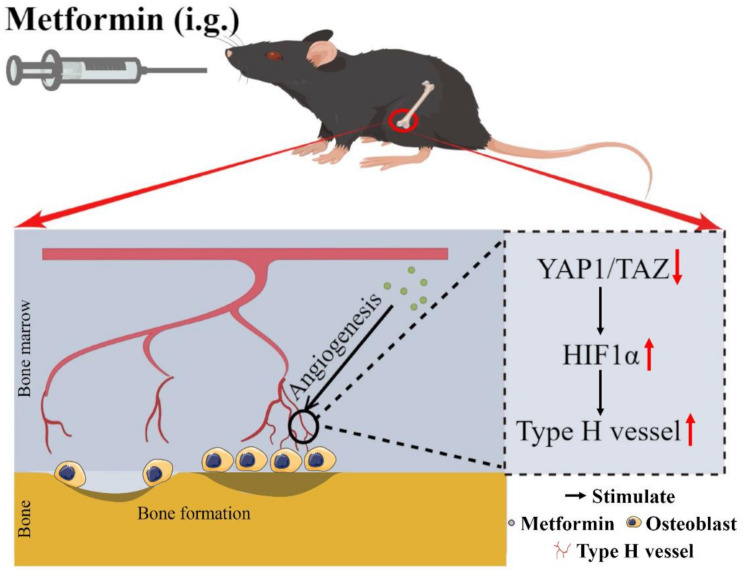

## Introduction

Osteoporosis is a chronic skeletal disease characterized by reduced bone mass and deteriorated bone microarchitecture, primarily affects postmenopausal women, the elderly, and astronauts (Sozen et al. 2017; Ensrud and Crandall 2017). Normal bone integrity is maintained by a coordinated balance of osteoblastic formation and osteoclastic resorption (Chotiyarnwong and McCloskey 2020). Additionally, it relies on the formation of blood vessels, which transport oxygen, nutrients, and growth hormones crucial for bone growth and remodeling (Grunewald et al. 2021; Watson and Adams 2018). In 2014, Adams et al. identified a unique vascular subtype in bone termed type H vessel, characterized by high expression of both CD31 and EMCN (CD31^hi^Emcn^hi^) (Kusumbe et al. 2014). These specialized blood vessels were found to mediate bone vasculature growth, sustain osteoprogenitors, and couple angiogenesis to osteogenesis (Kusumbe et al. 2014; Ramasamy et al. 2014). However, their abundance decreased with age in both animals and humans, accompanied by reduced osteoprogenitor counts and loss of bone mass (Kusumbe et al. 2014; Ramasamy et al. 2014; Wang et al. 2017). Subsequent studies by us and others revealed that type H vessels were also reduced in ovariectomized (OVX) and disuse mice (Xie et al. 2014; Liang et al. 2021). Our prior research demonstrated that SHP2 inhibitor NSC-87,877 exerts bone-protective effects by inhibiting osteoclast formation and increasing preosteoclast-induced platelet-derived growth factor-BB (PDGF-BB) production and type H vessel formation (Yin et al. 2018). Other factors such as Yes-associated protein 1 (YAP1)/Transcriptional activator with PDZ domain (TAZ) or hypoxia-inducible factor 1α (HIF1α), reported to affect type H vessel formation (Sivaraj et al. 2020), may also be potential targets for preventing and treating osteoporosis.

Transcriptional co-activators YAP and TAZ are critical components of the Hippo pathway, an evolutionarily conserved signaling cascade with pivotal roles in skeletal development, epithelial homeostasis, tissue regeneration, wound healing, and immune modulation (Yu et al. 2015). Increased expression and nuclear localization of YAP1/TAZ are associated with various diseases, notably cancer (Thompson 2020; Warren et al. 2018). However, the role of YAP1/TAZ in maintaining bone homeostasis remains controversial (Kegelman et al. 2020). HIF1α is one of the hypoxia-inducible factors, discovered as the regulatory factor adapting tissues or cells to hypoxia (Zhu et al. 2019). HIF1α is involved in numerous biological processes, including inflammation, glucose metabolism, and angiogenesis through modulation of integrin β2, glucose transporter, and vascular endothelial growth factor (Greer et al. 2012). Moreover, HIF1α can also enhance type H vessel formation (Kusumbe et al. 2014). A recent study demonstrated that YAP1/TAZ inhibits type H vessel formation by limiting the expression of HIF1α target genes in endothelial cells (ECs) (Sivaraj et al. 2020), prompting our investigation into whether a drug inhibiting/inactivating YAP1/TAZ could increase bone mass via HIF1α signaling-induced bone angiogenesis.

Metformin, a first-line medication for treating type 2 diabetes (Nasri and Rafieian-Kopaei 2014), has been studied for its therapeutic effects in non-diabetic conditions such as non-small-cell lung cancer, prostate cancer, cardiovascular diseases and osteoarthritis through regulating metabolic and cellular processes (Li et al. 2017, 2020; Yu et al. 2017; Castilla-Guerra et al. 2018; Blumel et al. 2020). While some studies have suggested that metformin reduces the risk of osteoporotic fracture and osteoporosis, its role and underlying mechanism in preventing and treatment remain unclear (Blumel et al. 2020; Tseng 2021; Liu et al. 2019; Jeyabalan et al. 2013). Metformin activates AMP-activated protein kinase (AMPK), which in turn inhibits YAP1/TAZ (Eyss et al. 2015). Therefore, metformin may exert bone-sparing effects by enhancing YAP1/TAZ-negatively-controlled type H vessel formation.

We hypothesized that metformin ameliorates osteoporosis by enhancing HIF1α signaling-induced bone angiogenesis via inhibiting YAP1/TAZ expression. To test this, we used different mouse models of osteoporosis, including early-stage OVX mice (one week after OVX, prevention) and late-stage OVX mice (eight weeks after OVX, treatment), as well as senile and disuse mice. Meanwhile, we employed human microvascular endothelial cells (HMECs) under normoxia (21% O_2_) and hypoxia (1% O_2_) conditions as a cell model. Our results demonstrate that metformin promotes bone mass and strength by increasing bone angiogenesis in osteoporotic mice. Mechanistically, metformin induces angiogenesis in vivo and in vitro through upregulation of HIF1α and its target genes, induced by downregulation of YAP1/TAZ. These findings suggest the potential utility of metformin in osteoporosis interventions.

## Results

### Metformin protects against bone loss in various osteoporosis mouse models

**Fig. 1:**
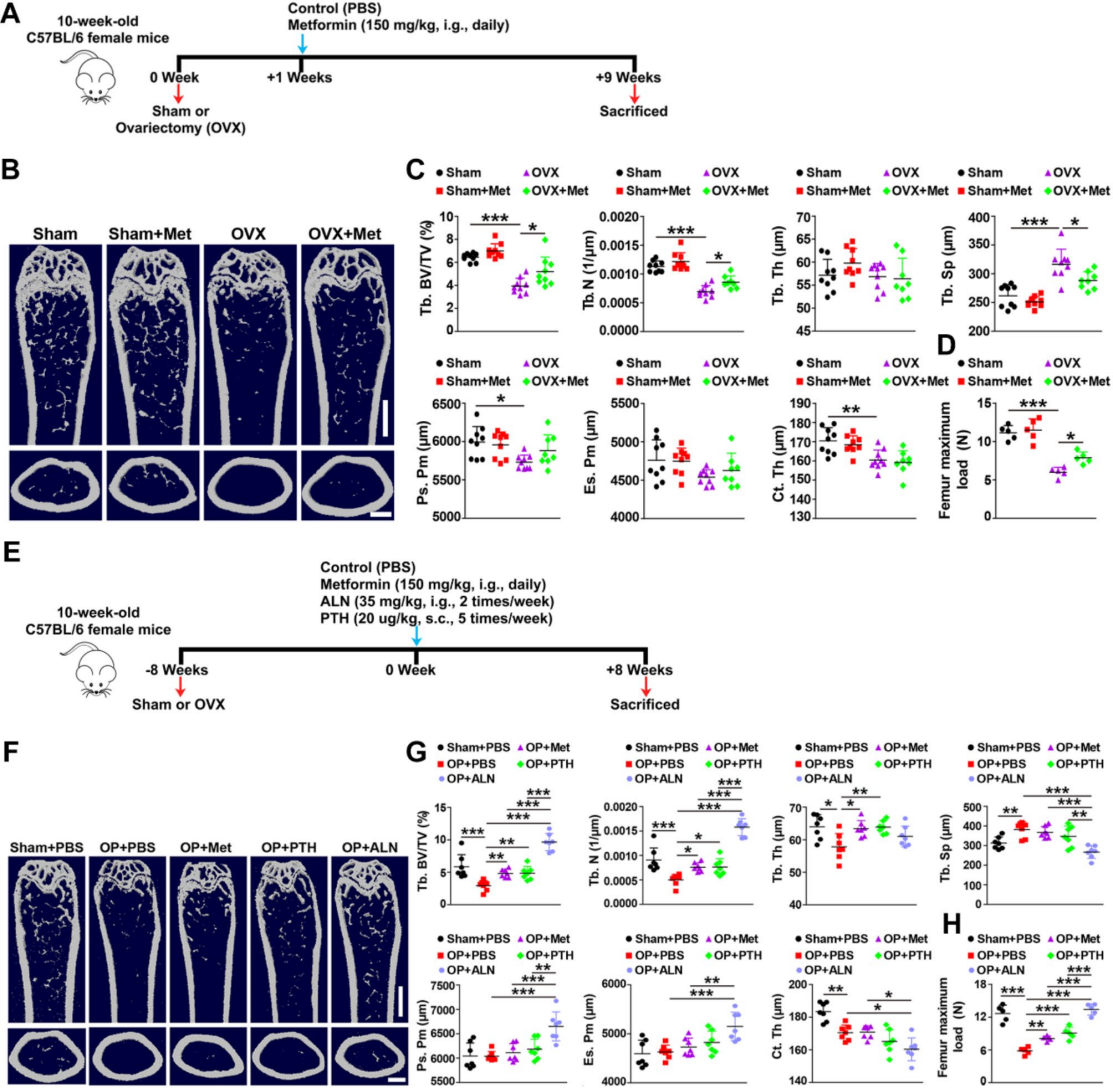
Metformin limits osteoporosis development and delays osteoporosis progression in OVX mice. (**A**) Experimental design for metformin treatment in OVX mice (one-week post-OVX). (**B**) Representative µCT images showing trabecular (up) and cortical (bottom) bone in femora from Sham, Sham + Metf, OVX, and OVX + Metf groups. µCT: microcomputed tomography. OVX: ovariectomized mice, seven days after ovariectomy mice. Metf: metformin. Scale bar: 2 mm (up) and 500 μm (bottom). (**C**) Quantitative analysis of trabecular bone volume fraction (Tb. BV/TV), trabecular number (Tb. N), trabecular thickness (Tb. Th), trabecular separation (Tb. Sp), periosteal perimeter (Ps. Pm), endosteal perimeter (Es. Pm) and cortical thickness (Ct. Th). *N* = 8–9 per group. (**D**) Three-point bending test of femur maximum load. *N* = 5 per group. (**E**) Experimental design for metformin, alendronate, and PTH treatment in long-term OVX mice (8 weeks post-OVX). (**F**) Representative µCT images showing trabecular (up) and cortical (bottom) bone in femora from Sham + PBS, OP + PBS, OP + Metf, OP + PTH and OP + ALN groups. OP: osteoporotic mice, two months after ovariectomy mice. PTH: parathyroid hormone. ALN: alendronate. Scale bar: 2 mm (up), 500 μm (bottom). (**G**) Quantitative analysis of Tb. BV/TV, Tb. N, Tb. Th, Tb. Sp, Ps. Pm, Es. Pm and Ct. Th. *n* = 7 per group; (**H**) Femur maximum load measured by three-point bending test. *n* = 5 per group. Data are plotted as mean ± SD. ^*^P < 0.05, ^**^P < 0.01, ^***^P < 0.001

**Fig. 2:**
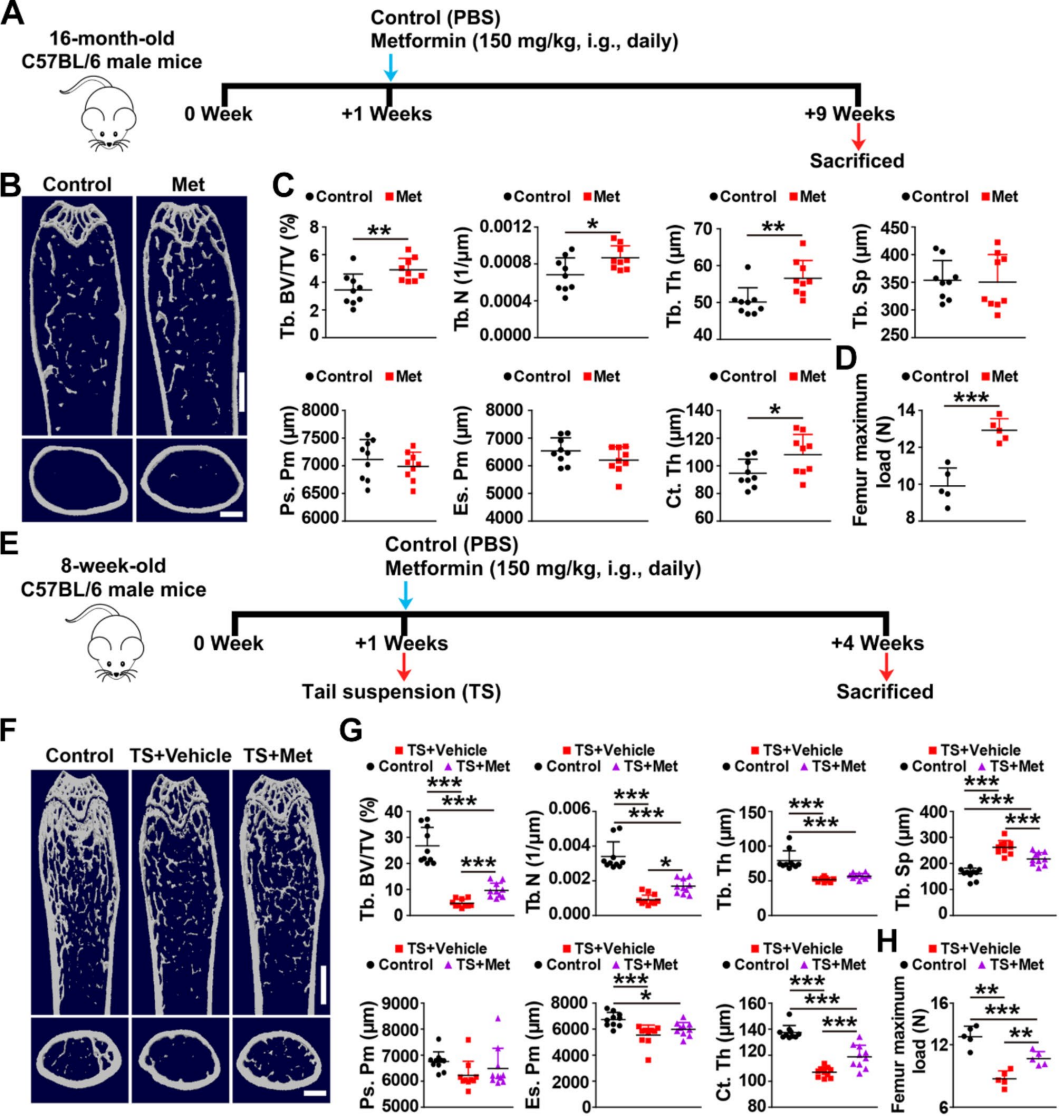
Metformin improves bone mass and strength in aged and tail-suspension mice. (**A**) Experimental design for metformin treatment in aged (16-month-old) mice. (**B-C**) Representative µCT image (**B**) and quantitative µCT analysis of trabecular (up) and cortical (bottom) bone microarchitecture (**C**) of femurs from PBS- (Control) and Metformin-treated aged mice. Scale bar: 2 mm (up) and 500 μm (bottom). *n* = 9 per group. (**D**) Three-point bending test of femur maximum load. *n* = 5 per group. (**E**) Experimental design for metformin treatment in tail-suspended (8-week-old) mice. (**F-G**) Representative µCT image (**F**) and quantitative µCT analysis of trabecular (up) and cortical (bottom) bone microarchitecture (**G**) in femora from Control, TS and TS + metformin mice. TS: tail-suspension. Scale bar: 2 mm (up), 500 μm (bottom). *n* = 10 per group; (**H**) Femur maximum load measured by three-point bending test. *n* = 5 per group. Data are plotted as mean ± SD. ^*^P < 0.05, ^**^P < 0.01, ^***^P < 0.001

The OVX animal serves as an experimental model of postmenopausal osteoporosis has been widely utilized (Habibi et al. 2022). To assess the effect of metformin on OVX-induced osteoporosis development and progression, we performed OVX in 10-week-old female C57/BL6J mice with or without metformin intervention, commencing either one week (prevention, named “OVX” in Fig. 1) or eight weeks (treatment, named “OP” in Fig. 1) after OVX. As shown in Fig. S1A-D, all OVX mice exhibited smaller uterus size and lower uterus weight compared to Sham-operated (Sham) mice, confirming the success of OVX. Subsequently, we conducted microcomputed tomography (µCT) analysis to evaluate bone mass and microarchitecture in the femora. As expected, femurs of OVX mice for the prevention experiment displayed notable osteoporotic phenotypes, including significantly decreased trabecular bone volume fraction (Tb. BV/TV), trabecular number (Tb. N), periosteal perimeter (Ps. Pm) and cortical thickness (Ct. Th), as well as increased trabecular separation (Tb. Sp) compared to Sham mice (Fig. 1A-C). Similarly, femurs of OVX mice for the treatment experiment showed lower Tb. BV/TV, Tb. N, trabecular thickness (Tb. Th) and Ct. Th, and higher Tb. Sp compared to Sham mice (Fig. 1E-G). Metformin intervention (150 mg/kg/day), commencing either one week or eight weeks after OVX, significantly enchanced bone mass and microarchitecture (Fig. 1B-C and F-G). However, metformin did not significantly impact bone mass and microarchitecture in Sham mice but had a promoting trend (Fig. 1B and C). As positive controls, PTH and ALN applied to the OVX mice for the treatment experiment, significantly ameliorated bone mass and microarchitecture (Fig. 1F and G). The three-point bending test revealed that the maximum load value, representing bone strength, was substantially lower in OVX mice compared to Sham mice, and metformin reversed this decrease in bone strength in OVX-induced osteoporotic mice (Fig. 1D and H). We further confirmed the therapeutic effects of metformin in senile and disused osteoporosis mouse models. µCT analysis and three-point bending tests uncovered that metformin significantly enhanced trabecular and cortical bone mass (Fig. 2A-C and E-G) and bone strength (Fig. 2D and H) in aged and tail-suspension (TS) mice.

**Fig. 3:**
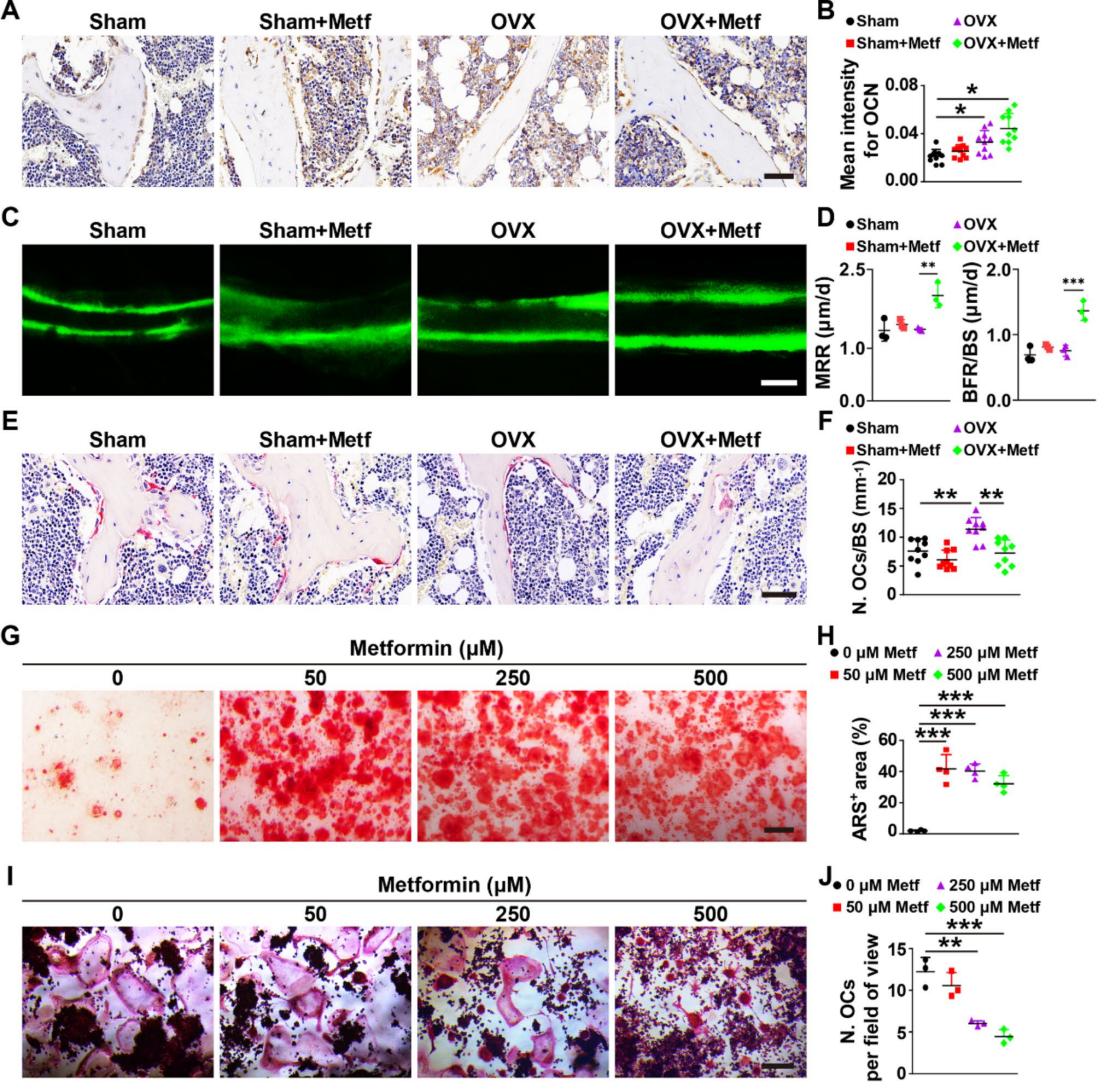
Metformin enhances osteogenesis and inhibits osteoclastogenesis in vivo and in vitro. (**A-B**) Representative OCN immunohistochemical staining images (**A**) with quantitative analysis of the mean intensity of OCN (**B**) in distal femora from Sham and OVX mice treated with vehicle or metformin. Scale bar: 50 μm; *n* = 10 per group. (**C-D**) Representative images of calcein double labeling of trabecular bone (**C**) with quantification of mineral apposition rate (MAR) and bone formation rate per bone surface (BFR/BS) (**D**). Scale bar: 10 μm. *n* = 3 per group. (**E-F**) Representative TRAP staining images (**E**) and quantitative analysis of osteoclast number (N. OCs; **F**) of trabecular bone surface (BS) in distal femora. Scale bar: 50 μm. *n* = 9 per group. (**G-H**) Representative Alizarin Red S (ARS) staining images (**G**) of mineralized nodules of BMSCs treated with metformin in different concentrations and quantitation of percentage of ARS positive areas (**H**) per field. Scale bar: 100 μm. *n* = 4 per group. (**I-J**) TRAP staining for osteoclast differentiation of RAW264.7 cells (**I**) and quantitation of the number of TRAP^+^ osteoclasts per field (**J**). Scale bar: 200 μm. *n* = 3 per group. Data are plotted as mean ± SD. ^*^P < 0.05, ^**^P < 0.01, ^***^P < 0.001

Next, we investigated the effect of metformin on osteogenesis and osteoclastogenesis in vivo and in vitro. Immunohistochemical staining for osteocalcin (OCN), a marker expressed in osteoblast, indicated a slightly increased mean intensity of OCN in OVX mice compared to Sham mice (Fig. 3A and B). In contrast, metformin intervention in OVX mice notably enhanced the mean intensity of OCN (Fig. 3A and B). Mineral apposition rate (MAR) and bone formation rate per bone surface (BFR/BS) measured by calcein double labeling suggested that metformin-treated OVX mice had a stronger ability to generate new mineralized bone compared to vehicle-treated mice (Fig. 3C and D). Tartrate-resistant acid phosphatase (TRAP) staining of femur sections showed that OVX mice have a larger number of osteoclasts surrounding trabecular bones compared to Sham mice, whereas treatment with metformin significantly reduced the osteoclast number in OVX mice (Fig. 3E and F). However, Sham mice treated with metformin exhibited only a trend towards promoting osteogenesis and inhibiting osteoclastogenesis compared with Sham mice treated with phosphate-buffered saline (PBS) (Fig. 3A-F). Metformin also enhanced the mean intensity of OCN (Fig. S2A) and reduced the number of osteoclasts (Fig. S2B) in trabecular bone of femora from the OVX mice in the treatment experiment, senile mice, and disused mice.

Subsequently, we investegated the effect of metformin on osteogenesis in bone marrow stromal cells (BMSCs) and osteoclastogenesis in RAW264.7 cells. Alizarin red S staining showed that metformin promoted calcium nodule formation of BMSCs in a concentration-dependent manner, especially at 250 µM (Fig. 3G-H). TRAP staining indicated that metformin reduced the number of osteoclasts during the osteoclastic differentiation of RAW264.7 cells (Fig. 3I-J). These findings demonstrate that metformin can prevent and treat osteoporosis by enhancing osteogenesis and inhibiting osteoclastogenesis.

### Metformin facilitates angiogenesis under hypoxia conditions in vivo and in vitro

**Fig. 4:**
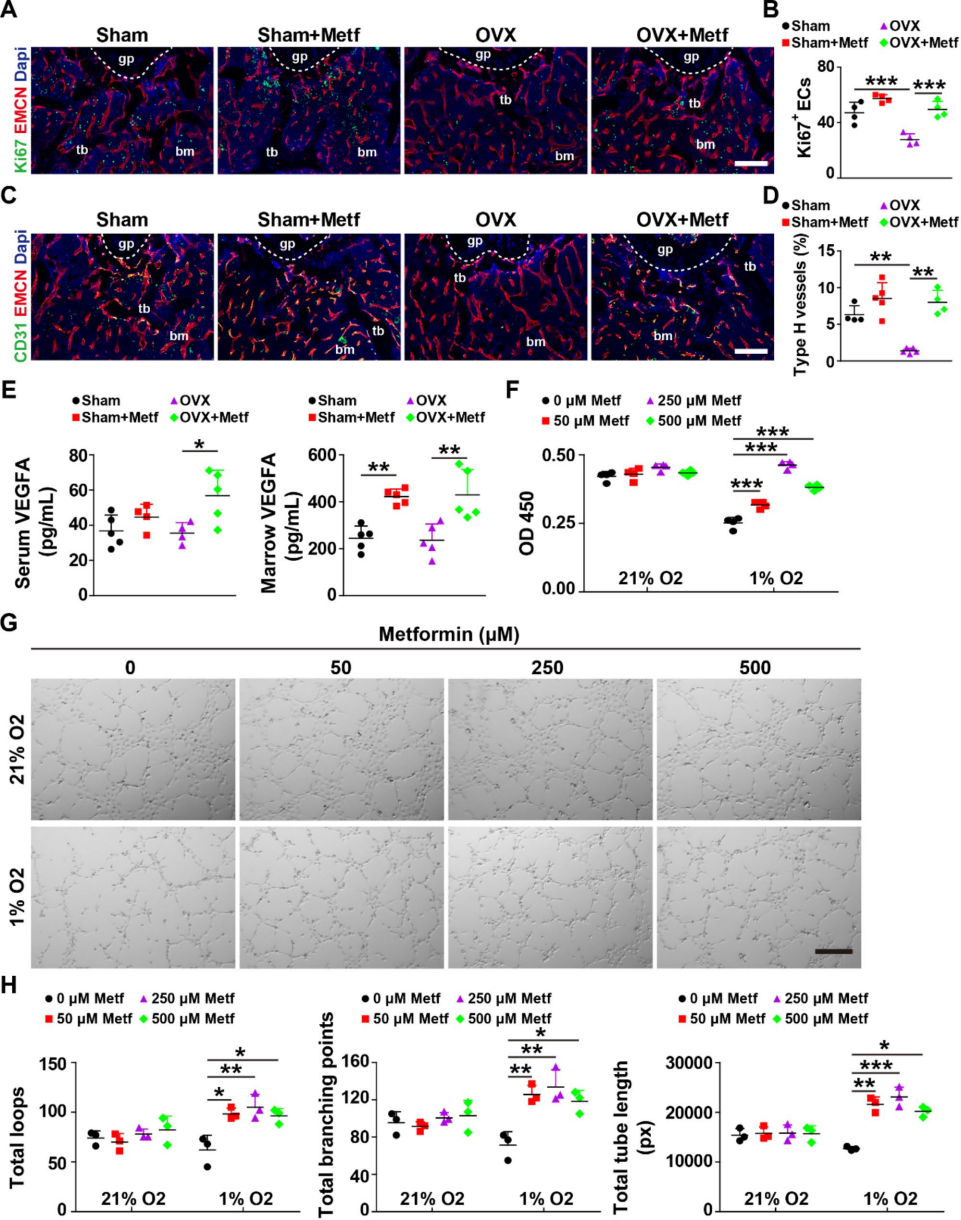
Metformin promotes type H vessel formation in OVX mice and elevates endothelial angiogenesis in vitro under hypoxic conditions. (**A-B**) Representative Ki67 and EMCN co-immunostaining images (**A**) with quantification of the number of Ki67 positive endothelial cells (Ki67^+^ ECs, **B**). gp: growth plate. tb: trabecular bone. bm: bone marrow. Scale bar: 200 μm. *n* = 4 per group. (**C-D**) Representative CD31 and EMCN co-immunostaining images (**C**) with quantification of the ratio of CD31^hi^EMCN^hi^ vessel (**D**) in femoral metaphysis from Sham, Sham + Metf, OVX and OVX + Metf groups. Scale bar: 200 μm. *n* = 4–5 per group. (**E**) ELISA for the serum and bone marrow concentrations of VEGFA. *n* = 4–5 per group; (**F**) CCK-8 analysis for the proliferation of metformin-treated HMECs under different oxygen concentrations (1% and 21% O_2_). *n* = 4 per group; (**G-H**) Representative images (**G**) of tube formation of HMECs on Matrigel with quantification of the total tube length, total loops and total branching points (**H**) in (G). Scale bar: 100 μm; *n* = 3 per group. Data are plotted as mean ± SD. ^*^P < 0.05, ^**^P < 0.01, ^***^P < 0.001

To confirm whether metformin can promote bone-specific angiogenesis, we performed Ki67 or CD31 co-immunostaining with EMCN on bone sections. As shown in Fig. 4A and B, the number of Ki67 positive endothelial cells (Ki67^+^ ECs) was significantly reduced in the metaphysis of femurs in OVX mice compared to Sham mice, and metformin administration to OVX mice notably increased the number of Ki67^+^ ECs. Consistent with our previous studies, OVX mice exhibited a lower percentage of type H vessels relative to Sham mice. Surprisingly, metformin significantly enhanced the percentage of this bone-specific vessel in OVX mice to a similar extent as in Sham mice (Fig. 4C and D). However, metformin had a moderate effect on type H vessel formation in the bone metaphysis of Sham mice, consistent with the trend of µCT data (Figs. 1A-C and 4E-G). ELISA analysis revealed that metformin remarkably elevated the concentrations of bone marrow VEGFA in both Sham and OVX mice but increased serum VEGFA concentrations in OVX mice (Fig. 4E). Inspired by Kishor K Sivaraj’s work (Sivaraj et al. 2020), we conducted a series of studies to determine whether metformin can promote angiogenesis under different oxygen concentration conditions in vitro. As shown in Fig. 4F, CCK-8 analysis revealed that metformin prominently promoted the proliferation of HMECs under hypoxia conditions, especially at 250 µM. The scratch wound assay demonstrated that metformin significantly promoted the mobility of HMECs under hypoxia conditions, with the most effective concentration being 250 µM (Fig. S3A and B). The tube formation assay on Matrigel is a cell model for verifying angiogenesis.

The tube formation assay on Matrigel, a cell model for verifying angiogenesis, showed notable increases in total tube length, total loops, and total branching points of metformin-treated HMECs under hypoxia conditions, especially at 250 µM (Fig. 4G and H). However, metformin did not affect the proliferation, migration, and tube formation of HMECs under normoxic conditions.

We also examined the percentage of type H vessels in the metaphysis of femurs from senile mice, OVX mice in the treatment experiment and TS mice. As shown in Fig. S4A and B, metformin-treated senile mice exhibited a considerably higher percentage of type H vessels compared to vehicle-treated senile mice. OVX mice in the treatment experiment and TS mice had a lower percentage of this bone-specific vessel compared to their control mice, whereas metformin reversed this effect (Fig. S4C and F). These results suggest that metformin enhances angiogenesis under hypoxia conditions in vivo and in vitro.

### Metformin increases HIF1α by inhibiting YAP1 and TAZ

**Fig. 5:**
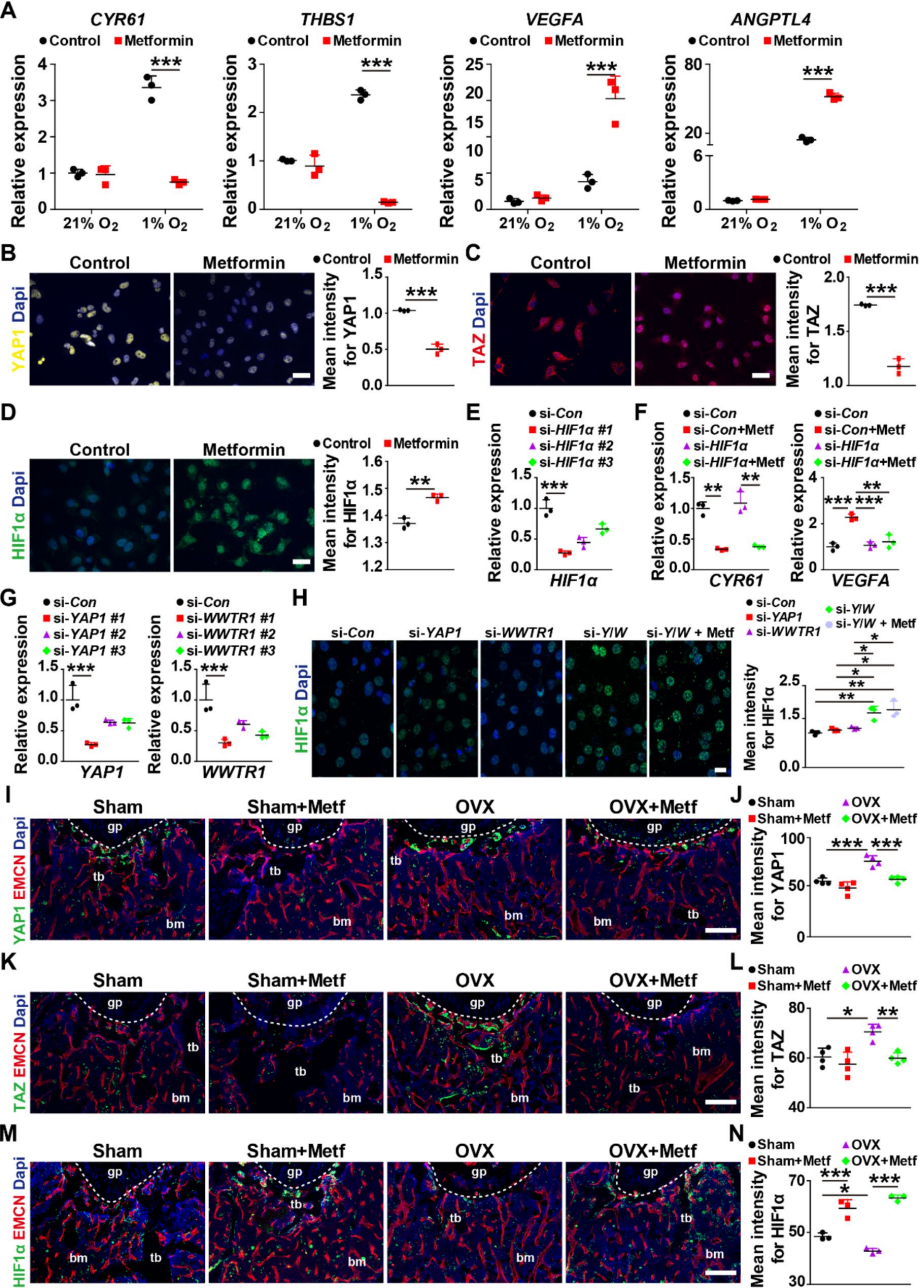
Metformin boosts the expression of HIF1α by inhibiting the expression of YAP1/TAZ. (**A**) qRT-PCR analysis for the mRNA level of the Yap1/Taz target genes *CYR61* and *THBS1* as well as the HIF1α target genes *VEGFA* and *ANGPTL4* in HMECs under different oxygen concentrations. *n* = 3 per group. (**B-D**) Immunofluorescence staining images and quantification showing the protein level of YAP1 (**B**), TAZ (**C**) and HIF1α (**D**) in HMECs treated with PBS (Control) or metformin under hypoxic conditions (1% O_2_). Scale bar: 20 μm. *n* = 3 per group. (**E**) qRT-PCR analysis showing the inhibitory efficiency of siRNAs targeting *HIF1α*. *n* = 3 per group. (**F**) qRT-PCR analysis of the expression of HIF1α target genes *Vegfa* and *Cyr61* in si-*HIF1α*-transfected HMECs under hypoxic conditions. *n* = 3 per group. (**G**) qRT-PCR analysis for the inhibitory efficiency of siRNAs targeting *YAP1* and *WWTR1*. *n* = 3 per group. (**H**) Immunofluorescence staining images and quantification showing the protein level of HIF1α in hypoxia-cultured HMECs from si-*Con*, si-*YAP1*, si-*WWTR1*, si-*Y*/*W* and si-*Y*/*W* + Metf groups. Y/W: *YAP1* and *WWTR1*. Scale bar: 20 μm. *n* = 3 per group. (**I-J**) Representative YAP1 and EMCN co-immunostaining images (**I**) with quantification of the mean intensity of YAP1 (**J**) in bone marrow from different groups. Scale bar: 200 μm. *n* = 4 per group. (**K-L**) Representative TAZ and EMCN co-immunostaining images (**K**) with quantification of the mean intensity of TAZ (**L**) in the bone marrow. Scale bar: 200 μm. *n* = 4 per group. (**M-N**) Representative HIF1α and EMCN co-immunostaining images (**M**) with quantification of the mean intensity of HIF1α (**N**). Scale bar: 200 μm. *n* = 3 per group. Data are plotted as mean ± SD. ^*^P < 0.05, ^**^P < 0.01, ^***^P < 0.001

Because metformin activates AMPK, which in turn inhibits the expression of YAP1/TAZ (Eyss et al. 2015), we investigated whether metformin can promote the expression of HIF1α and its target genes, which are positively related to type H vessel formation (Kusumbe et al. 2014), by reducing YAP1/TAZ expression. As shown in Fig. 5A, HMECs cultured under hypoxic conditions markedly upregulate YAP1/TAZ target genes *CYR61* and *THBS1*, as well as HIF1α target genes *VEGFA* and *ANGPTL4*, compared to normoxia conditions. However, the expression of *CYR61* and *THBS1* was strongly decreased, and the expression of *VEGFA* and *ANGPTL4* was significantly enhanced in metformin-treated HMECs under hypoxic conditions. No significant changes in these genes had been observed in metformin-treated HMECs versus PBS-treated HMECs under normoxia conditions, consistent with the above results of functional assays (Fig. 5A). Immunostaining confirmed that metformin significantly reduced the protein levels of YAP1 and TAZ and elevated the protein level of HIF1α in hypoxia-cultured HMECs (Fig. 5B-D). To investigate whether metformin-induced upregulation of VEGFA requires HIF1α, we used siRNA to interfere with *HIF1α* expression in hypoxia-cultured HMECs. Figure 5E showed that *si-HIF1α* #1 exhibited the highest silencing efficiency. si-*HIF1α* #1 and nontarget control siRNA (si-*Con*) was selected for subsequent experiments. As shown in Fig. 5F, metformin reduced the expression of the YAP1/TAZ target gene *CYR61* with or without si-*HIF1α* interference, whereas the HIF1α target gene *VEGFA* was no longer unregulated after si-*HIF1α* interfering in metformin-treated HMECs under hypoxic conditions, suggesting that metformin increases VEGFA expression by promoting HIF1α expression. To further confirm whether metformin upregulates HIF1α expression by inhibiting YAP1/TAZ in ECs under hypoxic conditions, we applied siRNA to interfere with *YAP1 and WWTR1* expression in hypoxia-cultured HMECs. Figure 5G showed that si-*YAP1* #1 and si-*WWTR1* #1 exhibited the highest silencing efficiency sequences. si-*YAP1* #1 and si-*WWTR1* #1 were thus selected for subsequent experiments. Immunostaining results revealed that either knockdown *YAP1* or *WWTR1* in hypoxia-cultured HMECs only slightly elevated the HIF1α expression, but knockdown both of *YAP1* and *WWTR1* markedly increased HIF1α expression, and metformin did not further increase this protein (Fig. 5H). These data confirm that metformin upregulates HIF1α and its target genes mainly through the inhibition of YAP1/TAZ expression.

We then performed immunostaining on bone sections from Sham, Sham + metformin, OVX and OVX + metformin mice and found that OVX mice had a higher level of YAP1/TAZ and a lower level of HIF1α compared to Sham mice, whereas metformin significantly decreased the expression of YAP1/TAZ and enhanced the expression of HIF1α in OVX mice (Fig. 5I-N). Moreover, metformin moderately decreased the YAP1/TAZ expression but significantly increased the HIF1α expression in Sham mice (Fig. 5I-N). These findings suggest that metformin can promote HIF1α expression by inhibiting the expression of YAP1 and TAZ in vivo and in vitro.

### Overexpression of YAP1/TAZ counteracts the bone-protective effect of Metformin

**Fig. 6:**
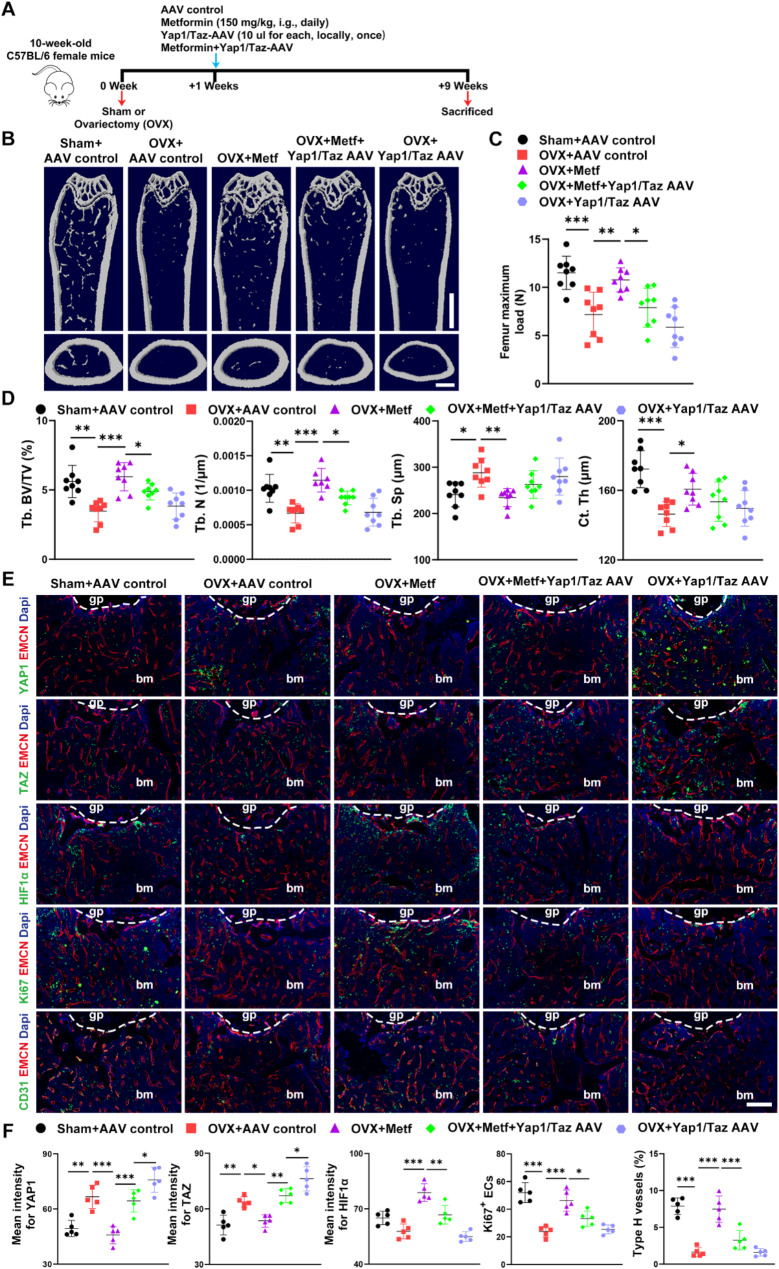
Overexpression of YAP1/TAZ counteracts the bone-protective effect of metformin. (**A**) Experimental design for metformin treatment and AAV-mediated YAP1/TAZ overexpression in OVX mice. (**B**) Representative µCT images of trabecular (up) and cortical (bottom) bone in femora from Sham + AAV control, OVX + AAV control, OVX + Metformin, OVX + Metformin + Yap1/Taz AAV and OVX + Yap1/Taz AAV groups. Scale bar: 2 mm (up), 500 μm (bottom). (**C**) Three-point bending measurement for maximum femur load from different groups. *n* = 8 per group. (**D**) Quantitative µCT analysis of Tb. BV/TV, Tb. N, Tb. Sp, and Ct. Th of trabecular and cortical bone in femora from OVX mice treated with different groups. *n* = 8 per group. (**E**) Representative images of YAP1, TAZ, HIF1α, Ki67 or CD31 co-immunostaining with EMCN in the metaphysis from different groups. Scale bar: 200 μm. (**F**) Quantification analysis of the mean intensity of YAP1, TAZ and HIF1α, the number of Ki67^+^ ECs and the ratio of CD31^hi^EMCN^hi^ vessel. *n* = 5 per group. Data are plotted as mean ± SD. ^*^P < 0.05, ^**^P < 0.01, ^***^P < 0.001

To verify whether metformin’s anti-osteoporotic effects depend on the YAP1/TAZ–HIF1α axis, we used an adeno-associated virus (AAV) to overexpress YAP1/TAZ in OVX mice and assessed its impact on metformin’s bone-protective benefits. OVX mice were divided into four groups: AAV Control, Metformin, YAP1/Taz AAV, and YAP1/Taz AAV + Metformin (Fig. 6A). Consistent with earlier findings, the three-point bending test demonstrated that metformin restored bone strength in OVX mice, while administering YAP1/Taz AAV alone produced only a mild decrease (Fig. 6C). µCT revealed that overexpressing YAP1/TAZ significantly attenuated metformin-induced increases in Tb. BV/TV and Tb. N, yet had no apparent effect on Tb. Sp or Ct. Th (Fig. 6B and D). Notably, Yap1/Taz AAV alone did not significantly alter bone mass or structure when compared to vehicle-treated OVX mice. Immunofluorescent staining of femoral metaphyses indicated that Yap1/Taz AAV markedly elevated YAP1/TAZ protein levels while reducing HIF1α, Ki67^+^ ECs, and type H vessels in metformin-treated OVX mice (Fig. 6E and F). Nevertheless, elevating YAP1/TAZ in OVX mice that did not receive metformin showed no discernible change in HIF1α expression, EC proliferation, or the proportion of type H vessels relative to vehicle-treated controls. Similarly, although Yap1/Taz AAV counteracted metformin’s osteogenic effects—evidenced by reduced OCN staining, MAR, and BFR/BS—it had no independent influence on bone formation (Fig. S5A and B). Finally, TRAP staining confirmed that YAP1/TAZ overexpression did not affect osteoclastogenesis in either OVX or metformin-treated OVX mice (Fig. S5C). These results suggest that the bone-protective effects of metformin may be partially attributed to increased HIF1α-dependent angiogenesis caused by decreased YAP1/TAZ.

## Discussion

Osteoporosis is associated with a high risk of fractures and poses a significant threat to the health and quality of life of aging populations, particularly postmenopausal women (Liu et al. 2021). While current therapies such as bisphosphonates and denosumab are effective (Gorai 2015), they are associated with substantial side effects such as atypical femoral fractures and jaw osteonecrosis, necessitating the exploration of alternative treatments (Black et al. 2012; Cosman et al. 2016). As a classic anti-diabetic drug, metformin is associated with a lower risk of osteoporosis in adult women independent of type 2 diabetes mellitus and obesity (Blumel et al. 2020). However, previous studies have yielded conflicting results regarding the effects of metformin on bone mass in animal models (Liu et al. 2019; Zhao et al. 2019; Zhou et al. 2022). Here, we demonstrate that intragastric administration of metformin effectively induces bone-protective effects in osteoporotic mice.

Angiogenesis plays a critical role in maintaining bone homeostasis (Diomede et al. 2020), as bone formation relies on the development of new blood vessels and the molecular signals derived from endothelial cells (Huang et al. 2018). Type H vessels are particularly crucial in governing bone vasculature formation, provide unique metabolic and molecular microenvironments, maintaining perivascular osteoprogenitors, and couple angiogenesis to osteogenesis (Yang et al. 2017). Thus, increasing the formation of type H vessels presents a promising strategy for preventing and treating bone loss. Our study, along with others, has demonstrated that the reduction in the abundance of this distinct vessel subtype is associated with bone loss in various conditions, including OVX-, aging- or disuse-induced osteoporosis in mice (Kusumbe et al. 2014; Liang et al. 2021; Yang et al. 2017). Moreover, compounds such as harmine emulsion, NSC-87,877, and Panax quinquefolium saponin have been shown to reverse bone loss caused by the reduced of type H vessels (Liang et al. 2021; Yin et al. 2018; Huang et al. 2018).

In our study, we observed that metformin promoted angiogenesis in HMECs cultured under hypoxia conditions but had no effect on angiogenesis under normoxia conditions. Given the presence of extensive hypoxic areas in postnatal long bones (Sivaraj et al. 2020), we then investigated whether metformin could enhance bone angiogenesis in vivo. Surprisingly, we found that metformin significantly boosted the ratio of CD31^hi^EMCN^hi^ vessels in the metaphysis of osteoporotic mice. Additionally, previous research by Sivaraj et al. indicated that YAP1/TAZ inhibits angiogenesis in anoxic bone tissue by limiting hypoxia-inducible factor signaling, in contrast to their pro-angiogenic role in other organs (Sivaraj et al. 2020). Considering that metformin can activate AMPK, which in turn inhibits the expression of YAP1 and TAZ (Eyss et al. 2015), it is plausible that these transcriptional co-activators play a crucial role in metformin-stimulated bone angiogenesis.

The transcriptional co-activators YAP1/TAZ are the critical components of Hippo signaling, which is essential for vasculature growth (Hermann et al. 2021). Deletion of Yap1/Taz in ECs has been shown to inhibit endothelial proliferation and sprouting in embryo and postnatal retina (Kim et al. 2015, 2017). Recent studies have indicated that the inactivation of YAP1/TAZ in ECs enhances the number of CD31^hi^EMCN^hi^ vessels in the hypoxic bone tissue while suppressing vascular growth in other organs through HIF signaling (Sivaraj et al. 2020). HIF1α, a crucial transcription factor in the hypoxia-inducible factor pathway, plays an essential role in bone angiogenesis by regulating the formation and maintenance of type H endothelial cells, thereby coupling angiogenesis to osteogenesis (Kusumbe et al. 2014, 2016). Our findings demonstrate that metformin inhibited the expression of YAP1/TAZ and enhanced the expression of HIF1α in HMECs cultured under hypoxic conditions and in the bone metaphysis. Moreover, we employed AAV to overexpress YAP1/TAZ locally in OVX mice. Overexpression of YAP1/TAZ partially reversed metformin’s pro-angiogenic and osteogenic effects, resulting in lower levels of HIF1α, fewer type H vessels, and diminished trabecular bone volume. By contrast, YAP1/TAZ overexpression alone did not significantly affect bone parameters in untreated OVX mice, indicating that metformin’s effect is indeed contingent upon suppressing YAP1/TAZ.

Bone remodeling is tightly regulated by bone-forming osteoblasts and bone-resorbing osteoclasts (Weivoda et al. 2020). In our study, we found that metformin significantly increased osteogenesis and decreased osteoclastogenesis in vitro and in vivo. Although the precise mechanism whereby metformin inhibits osteoclastogenesis requires further investigation, it likely reflects both local and systemic metabolic changes induced by AMPK activation. Moreover, because YAP1/TAZ have been implicated in controlling bone cell differentiation and function (Kegelman et al. 2018, 2021; Xiong et al. 2018), it is plausible that metformin’s suppression of YAP1/TAZ indirectly influences osteoclasts via crosstalk among osteoblasts, osteoclasts, and the bone vasculature. Future experiments using cell-specific YAP1/TAZ gain-of-function or loss-of-function mouse models will help clarify these interactions more thoroughly.

In conclusion, our findings demonstrate that metformin administration protects bone by promoting type H vessel formation and angiogenesis in osteoporotic mice. The molecular mechanism appears to involve inhibiting YAP1/TAZ and thereby upregulating HIF1α, which promotes healthy bone vasculature. By employing AAV-based YAP1/TAZ overexpression, we have confirmed that reducing YAP1/TAZ levels is a key component of metformin’s effect on bone angiogenesis and osteogenesis. These results underscore metformin’s potential as a clinically relevant treatment option for osteoporosis and highlight the YAP1/TAZ–HIF1α axis as a promising therapeutic target in future bone research.

## Materials and methods

### Animals and treatments

Approval for this study was obtained from the Ethics Committee of Xiangya Hospital of Central South University (**No. 202008010**). Animal care and experiments were conducted following the guidelines of the Department of Laboratory Animals of Central South University. To investigate the effects of metformin on postmenopausal osteoporosis, 10-week-old C57BL/6 female mice (22–25 g) underwent either bilateral ovariectomy or a sham operation (Chen et al. 2019). One week or eight weeks after surgery, mice were treated daily by oral gavage with metformin (150 mg/kg/day) or an equal volume of PBS. To further examine the role of YAP1/TAZ in metformin-mediated bone protection, we utilized an AAV system to overexpress YAP1/TAZ in vivo. OVX mice were divided into four groups: (Sozen et al. 2017) OVX + AAV Control (Ensrud and Crandall 2017), OVX + Metformin (Chotiyarnwong and McCloskey 2020), OVX + Yap1/Taz AAV, and (Grunewald et al. 2021) OVX + Metformin + Yap1/Taz AAV. AAV injections were performed under anesthesia, with 10 µL of either control AAV or Yap1/Taz-AAV (1 × 10¹² viral particles/mL) administered locally into the distal femoral metaphysis. All mice were euthanized two months later, and serum, uteri, tibial bone marrow, and femora were collected for subsequent analyses. For the senile osteoporosis model, 16-month-old C57BL/6 male mice received metformin (150 mg/kg/day) or PBS five times weekly for two months. In the disuse osteoporosis model, 10-week-old male mice were subjected to hindlimb unloading by TS and treated with metformin (150 mg/kg/day) or PBS five times weekly for three weeks. These experimental protocols ensure consistency across models while allowing for a systematic evaluation of metformin’s effects on bone mass, strength, and angiogenesis under different osteoporotic conditions.

### Μicro-CT analysis

Femora fixed in 4% paraformaldehyde for 48 h, and then scanned (voltage: 70 kV; current: 400 µA; X-ray tube potential: 55 kVp; integration time: 400 ms; voxel size: 11.4 μm) by vivoCT80 (SCANCO Medical AG; Brusttisellen, Switzerland). Images were analyzed and visualized using data analysis software (CTAn v1.11) and three-dimensional model visualization software (µCTVol v2.2). The region of interest (ROI) of trabecular bone was the area between 0.45 mm and 0.90 mm proximal to the growth plate in the distal femurs. Tb. BV/TV, Tb. N, Tb. Th and Tb. Sp were measured. For cortical bone, the ROI selected for scanning was 10% of femoral length in mid-diaphysis of the femur. Es. Pm, Ps. Pm and Ct. Th were assessed.

### Biomechanical test

The Instron 3343 (Instron, Canton, USA) was used to perform a three-point bending test to assess bone strength. The loading point is located in the middle of the femur, with two fulcrums 8 mm apart and a loading speed of 5 mm/min. The load-deformation curves were used to collect biomechanical measurement data. The femur’s ultimate load value (*N*) was calculated and recorded.

### Histological, immunohistochemical and histomorphometric analyses

To conduct histological and immunohistochemical investigations, femora were dissected and fixed in 4% paraformaldehyde for 48 h, decalcified in 18% EDTA (pH = 7.4) for one week, dried using graded ethanol of increasing concentration, and embedded in paraffin. As previously described (Chen et al. 2019), samples were cut into 5-m-thick longitudinally oriented bone sections and stained with OCN and TRAP. The images were taken using an optical microscope (CX31; Olympus, Hamburg, Germany). For OCN and TRAP staining, positively stained cells (osteoblasts and osteoclasts) were counted in three random visual fields per section. The number of neighboring-bone-surface osteoclasts per millimeter (N/mm) and the mean intensity for OCN were quantified. Abcam (Cambridge, Britain) provided the anti-OCN and all secondary antibodies. Sigma (St. Louis, MO, USA) provided the TRAP staining kit.

To assess dynamic bone formation, mice were injected intraperitoneally with 0.1% calcein (10 mg/kg body weight; Sigma) in PBS ten and three days before euthanasia. Femora were collected, fixed in 4% paraformaldehyde for 48 h, and dehydrated in ethanol concentrations ranging from 0 to 100%. These bones that remained undecalcified were implanted in methyl methacrylate. A microtome was used to slice the femur into 60 μm slices. The Leica fluorescent microscope was used to analyze the double labeling of calcein. The MAR and BFR/BS of trabecular bone were determined using Image-Pro Plus 6 software in three randomly selected visual areas in the distal metaphysis of each femur slice.

### Culture of BMSCs and RAW264.7

BMSCs were obtained from the bone marrow of C57BL/6 female mice’s femurs and tibias as previously described (Wang et al. 2022). The American Type Culture Collection (Rockville, MD, USA) provided the osteoclast progenitor RAW264 cells (Rockville, MD, USA). All cells were grown in α-MEM (Gibco, Grand Island, USA) supplemented with 10% fetal bovine serum (FBS; Gibco), 100 units per milliliter penicillin (Gibco), and 100 units per milliliter streptomycin (Gibco) (Gibco). Cells were maintained at 37 ℃ in a humidified atmosphere containing 5% CO_2_ and passaged when they reached 80% confluence.

### Osteogenic differentiation assay

In 48-well plates, BMSCs were seeded and cultured in α-MEM containing 10% FBS. When cells reached 100% confluence, osteogenic differentiation was induced using a commercially available osteogenic induction kit (MUXMX-90021, Cyagen Biotechnology, China), following the manufacturer’s protocol. The induction medium contained α-MEM, 10% FBS, 50 µg/mL ascorbic acid, 10 mM β-glycerophosphate, and 100 nM dexamethasone. To assess the effect of metformin, the medium was supplemented with 0–500 µM metformin or an equal volume of PBS as a vehicle control. BMSCs cultured in α-MEM supplemented with 10% FBS were used as a negative control. Every alternate day, half of the medium was replaced. After 9 days of osteogenic differentiation, the cultures were rinsed with PBS, fixed for 5 min with 4% paraformaldehyde, and stained with ARS solution (Solarbio, Beijing, China) to assess cell-matrix mineralization.

### Osteoclastic differentiation assay

RAW264.7 cells were seeded in 48-well plates at a density of 10^4^ cells per well and incubated overnight. After washing the cells with PBS, the media was replaced with fresh complete DMEM supplemented with 100 ng/mL RANKL and 0-500 µM metformin, or an equal volume of PBS. The negative control culture was grown in full DMEM supplemented with the same volume of vehicle as the positive control culture. Every other day, half of the medium was replaced. After 8 days of induction, the cells were stained for TRAP using a commercially available kit (Sigma) following 10 min of fixation in 4% paraformaldehyde and washing with PBS. Osteoclasts were defined as TRAP positive multinucleated cells with more than three nuclei. Under inverted microscopy (Leica), the number of osteoclasts per field of view was counted.

### Immunofluorescent analyses

For bone immunofluorescent analyses, the femora were promptly fixed in ice-cold 4% paraformaldehyde for 4 h, decalcified in 18% EDTA for 3 days, dried in 30% sucrose, and then embedded in OCT. The samples were sliced longitudinally into 30 μm-thick pieces of bone. Following that, bone slices were incubated overnight at 4 ℃ with Ki67 (Servicebio, China), CD31 (Abcam, Britain), EMCN (Santa Cruz, USA), YAP1 (Cell Signaling Technology, USA), TAZ (Atlas Antibodies, Sweden), or HIF1α (Invitrogen, USA) antibodies, followed by 1 h at room temperature with secondary antibodies. As negative controls, sections treated with secondary antibodies alone were employed. DAPI (Invitrogen, USA) was used to stain the nuclei.

For cell immunofluorescent staining, HMECs were washed with ice-cold PBS and fixed in 4% paraformaldehyde for 15 min, and then these cells were incubated in 0.1% Triton in PBS for 15 min to permeate the cell membranes. After washing twice with PBS, the cells were blocked with PBS containing 5% FBS for 15 min at 4 ℃. The cells were incubated overnight at 4 °C with YAP1 (Cell Signaling Technology, USA), TAZ (Atlas Antibodies, Sweden), and HIF1α (Invitrogen, USA) antibodies, followed by secondary antibody incubation.

All secondary antibodies were obtained from Abcam (Jackson Immuno Research, USA). All images were obtained under a Zeiss ApoTome fluorescence microscope (Jena, Germany). The ratio of CD31^hi^EMCN^hi^ vessels in EMCN positive vessels (%), the number of Ki67 positive endothelial cells (Ki67^+^ ECs), and the mean intensity of YAP1, TAZ and HIF1α were calculated.

### ELISA

The serum and bone marrow samples were collected and frozen at − 80 °C until used in the test. According to the manufacturer’s instructions, the concentrations of VEGFA were determined by commercial Mouse VEGF ELISA Kits (Multi Sciences LTD., Hangzhou, China). Each well’s optical density was measured with a microplate reader (Bio-Rad 680, Hercules, USA) set to 450 nm at a wavelength of 570 nm. Each sample’s protein concentration was determined using the standard curve.

### Tube formation assay

HMECs were seeded at a density of 1 × 10^4^ cells per well onto Matrigel-coated 96-well plates and treated with 0-500 M metformin under normoxia (21% O_2_) or hypoxia (1% O_2_) conditions. Tube formation was discovered after 6 h using an inverted microscope (Leica). A blinded independent observer counted the total branching points, total tube length, and total loops per image.

### Migration assay

HMECs (2 × 10^5^ per well) were plated into a 12-well plate and cultured at 37 °C for the scratch wound assay. After cells had adhered, the monolayer was scraped and rinsed with PBS to remove unattached cells. The cells were then treated with various amounts of metformin in normoxia (21% O_2_) or hypoxia (1% O_2_) conditions. At 0 h and 24 h post-wounding, cells were imaged. The migration area rate was computed as previously described (Yin et al. 2018): Migration area (%) = (A_0_– An)/A_0_ × 100, where A_0_ is the beginning wound area and An is the remaining wound area at the metering point.

### Cell proliferation assay

The proliferation of HMECs was determined using the cell counting kit-8 test (CCK-8; Dojindo, Kumamoto, Japan). Cells were seeded at a density of 5 × 10^3^/well in 96-well plates and treated with metformin at various doses in normoxia (21% O_2_) or hypoxia (1% O_2_). After 48 h, the culture media were supplemented with CCK-8 solution (10 µL per well), and cells were incubated at 37 °C for 3 hours. The absorbance at 450 nm was determined using a microplate reader (Bio-Rad 680), and proliferation was calculated as the mean absorbance of each well minus the blank value.

### qRT-PCR analysis

Total RNA was isolated using the TRIzol Reagent (Invitrogen) and reverse transcribed into cDNA using the RevertAid First Strand cDNA Synthesis kit (Fermentas, Burlington, Canada). Subsequently, cDNA was amplified on an ABI PRISM^®^ 7900HT System (Applied Biosystems, Foster City, USA) using FastStart Universal SYBR Premix ExTaqTM II (Takara Biotechnology, Japan). The 2^–ΔΔCT^ method was employed to quantify relative gene expression, and GAPDH was used as a housekeeping gene for internal standardization. The primer sequences used for qRT-PCR are shown in Supplementary Table 1.

### RNA interference

These siRNAs, including *YAP1* (si-*YAP1* #1, 2 and 3), *WWTR1* (si-*WWTR1* #1, 2 and 3) and *HIF1α* (si-*HIF1α* #1, 2 and 3), were purchased from RiboBio (Guangzhou, China). Cells transfection was performed according to the instructions of the manufacturers. In brief, HMECs cells were transfected with si-*YAP1*, si-*WWTR1*, si-*HIF1α* or Con siRNA using Lipofectamine 2000 (Invitrogen, Carlsbad, CA). 48 h later, the inhibitory efficiency of these siRNAs was verified by quantitative real-time PCR (qRT-PCR) analysis. The most effective siRNAs were used for further investigating. The target sequences of these siRNAs are shown in Supplementary Table 2.

### Statistical analysis

Data are presented as mean ± standard deviation (SD). The unpaired, two-tailed Student’s t-test was used to determine differences between two groups. Multiple group comparisons were performed using one-way analysis of variance (ANOVA) and the Bonferroni post hoc test to establish the significance of differences between two groups. These analyses were conducted using the GraphPad Prism software, and statistically significant differences were defined as those with *P* < 0.05.

## Electronic supplementary material

Below is the link to the electronic supplementary material.

Supplementary Figures: 1-5 and Tables 1-2.

## Data Availability

All the data are available from the corresponding author on reasonable request.
