# Supplementary material for: Metformin ameliorates osteoporosis by enhancing bone angiogenesis via the YAP1/TAZ-HIF1α axis
Source: Mol Med. 2025 Mar 30;31:122. doi: 10.1186/s10020-025-01169-7 (PMC11955141; doi:10.1186/s10020-025-01169-7)
Supplement: Supplementary file 1 — Supplementary Figures: 1-5 and Tables 1-2. [file 10020_2025_1169_MOESM1_ESM.docx]

**Supplemental information**

**Metformin ameliorates osteoporosis by enhancing bone angiogenesis via the**

**YAP1/TAZ-HIF1α axis**

Hao Yin^#^, Zhe Ruan^#^, Teng-Fei Wan, Zhi-Rou Lin, Chun-Yuan Chen, Zhen-Xing Wang, Jia Cao, Yi-Yi Wang, Ling Jin, Yi-Wei Liu, Guo-Qiang Zhu, Jiang-Shan Gong, Jing-Tao Zou, Yi Luo, Yin Hu, Zhao-Hui Li, Hao Luo, Yu-Qi Liu, Cheng Long, Shu-Shan Zhao**^*^**, Yong Zhu**^*^**, Hui Xie**^*^**

***** Corresponding authors: Hui Xie (huixie@csu.edu.cn); Yong Zhu (doczhu2003@aliyun.com); Shushan Zhao (shushanzhao@csu.edu.cn).


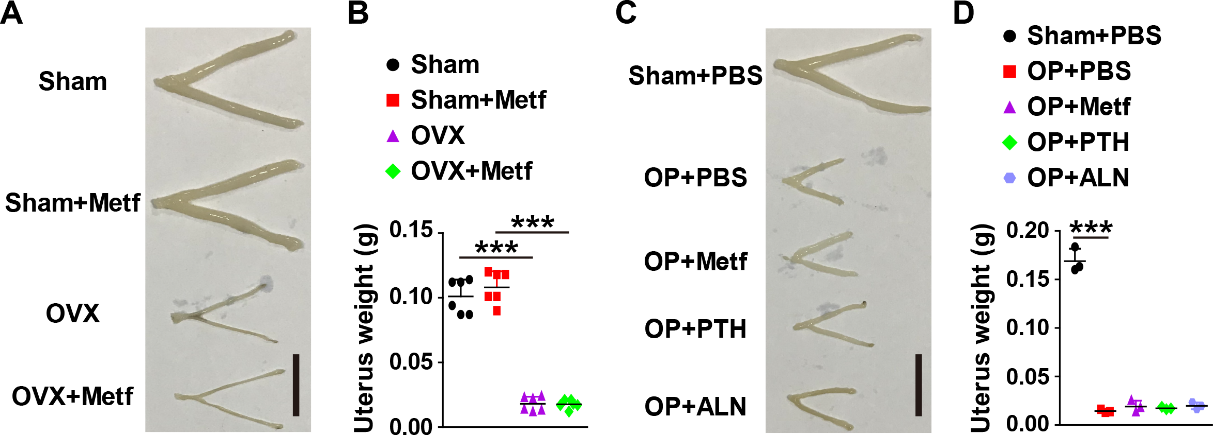


**Figure. S1 OVX mice display smaller uterus size and lower uterus weight.**

**(A-B)** Representative images **(A)** of uterus size with quantification analysis of uterus weight **(B)** from Sham and OVX mice treated with PBS or metformin. Scale bar: 1 cm. n = 6 per group. **(C-D)** Representative uterus images **(C)** with quantification analysis of uterus weight **(D)** from Sham + PBS, OP + PBS, OP + Metf, OP + PTH and OP + ALN mice. Scale bar: 1 cm. n = 3 per group. Data are plotted as mean ± SD. ^*^*P* < 0.05, ^**^*P* < 0.01, ^***^*P* < 0.001.


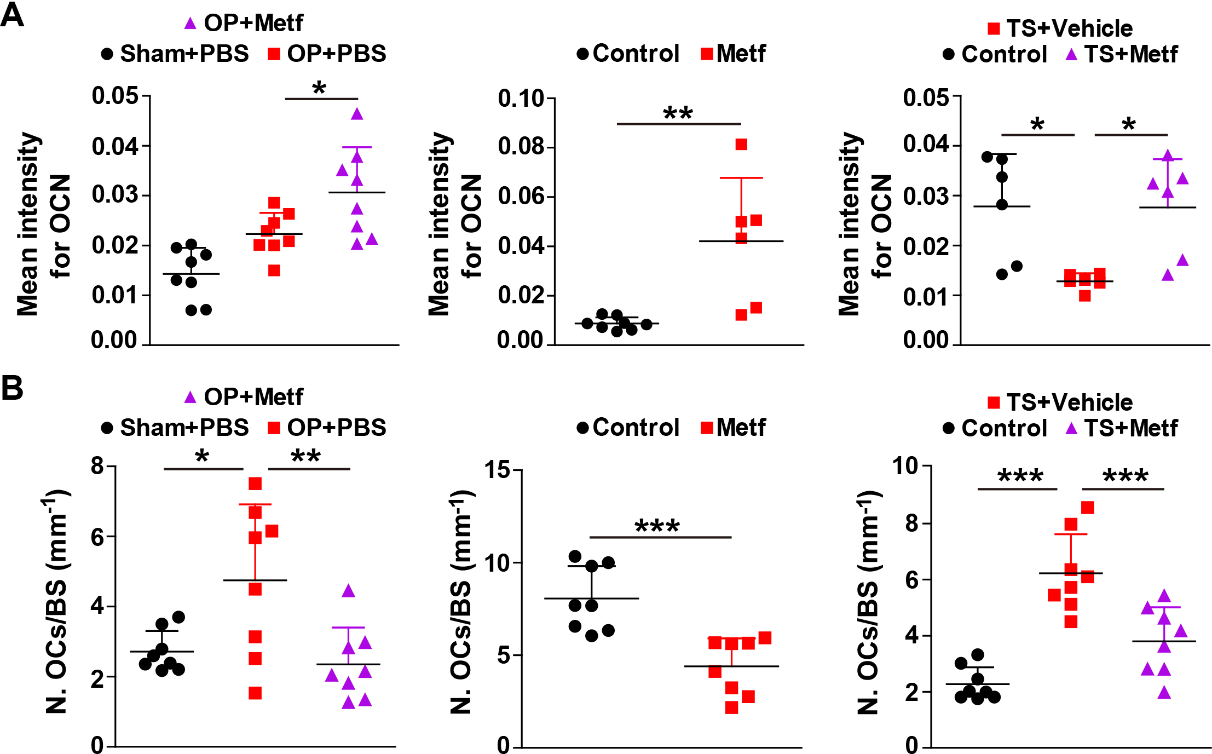


**Figure. S2 Metformin augments osteogenesis and inhibits osteoclast formation** **in multiple mouse models of osteoporosis.**

**(A)** Quantification analysis of the mean intensity of OCN in distal femora from OVX-, senile- and TS-induced osteoporotic mice. n = 6-8 per group. **(B)** Quantitative analysis of osteoclast number (N. OCs) of trabecular bone surface (BS) in femora from OVX-, senile- and TS-induced osteoporotic mice. n = 8 per group. Data are plotted as mean ± SD. ^*^*P* < 0.05, ^**^*P* < 0.01, ^***^*P* < 0.001.


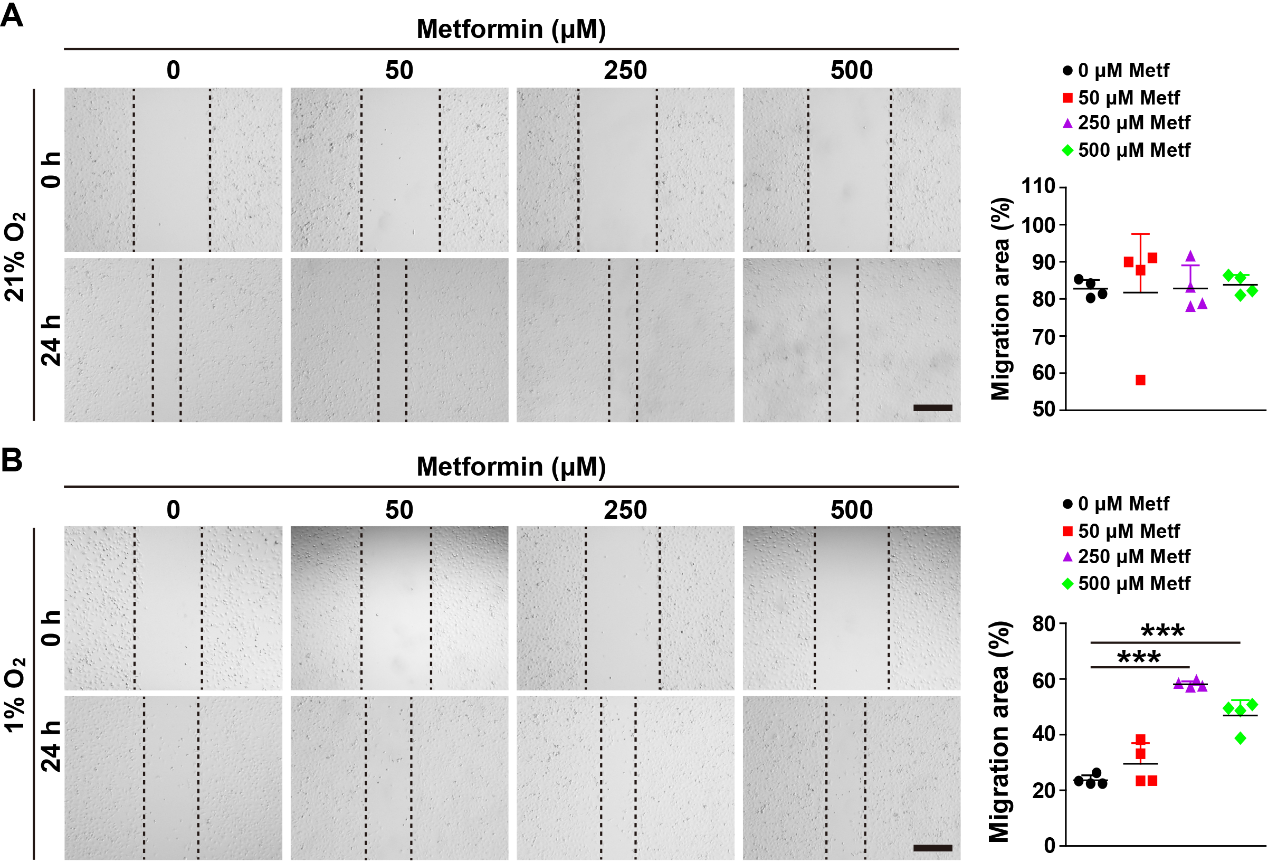


**Figure. S3 Metformin promotes the migration of HMECs under hypoxic conditions.**

**(A-B)** The migration of metformin-treated HMECs under normoxia **(A)** and hypoxic conditions **(B)** measured by the scratch wound assay. Scale bar: 200 μm. n = 4 per group. Data are plotted as mean ± SD. ^*^*P* < 0.05, ^**^*P* < 0.01, ^***^*P* < 0.001.


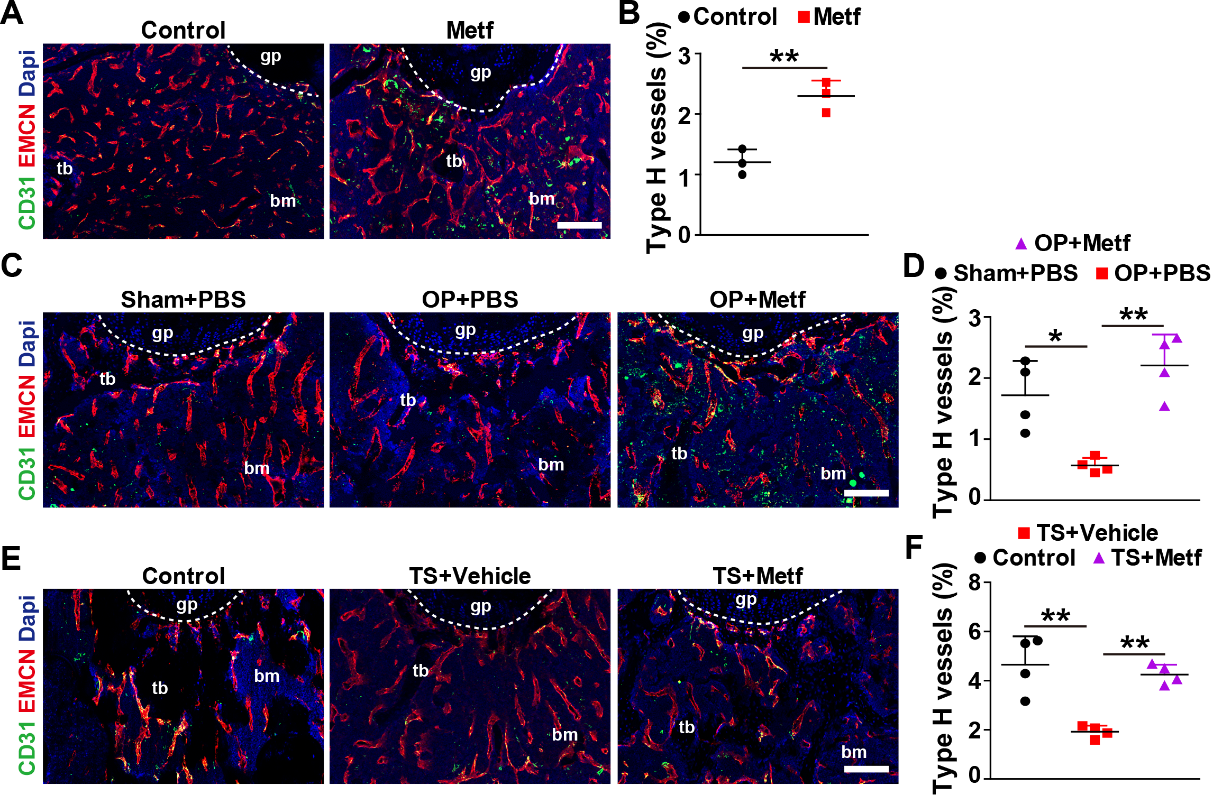


**Figure. S4 Metformin facilitates type H vessel formation in multiple mouse models of osteoporosis.**

**(A-B)** Representative CD31 and EMCN co-immunostaining images **(A)** with quantification of the ratio of type H vessel **(B)** in femoral metaphysis from PBS- (Control) and metformin-treated aged mice. Scale bar: 200 μm. n = 3 per group. **(C-D)** Representative CD31 and EMCN co-immunostaining images **(C)** and quantified the ratio of type H vessel **(D)** in femoral metaphysis from Sham + PBS, OP + PBS and OP + Metf mice. Scale bar: 200 μm. n = 4 per group. **(E-F)** Representative images **(E)** of CD31 and EMCN co-immunostaining and quantification of type H vessel ratio **(F)** in femoral metaphysis from Control, TS + Vehicle and TS + Metf mice. Scale bar: 200 μm. n = 4 per group. Data are plotted as mean ± SD. ^*^*P* < 0.05, ^**^*P* < 0.01, ^***^*P* < 0.001.


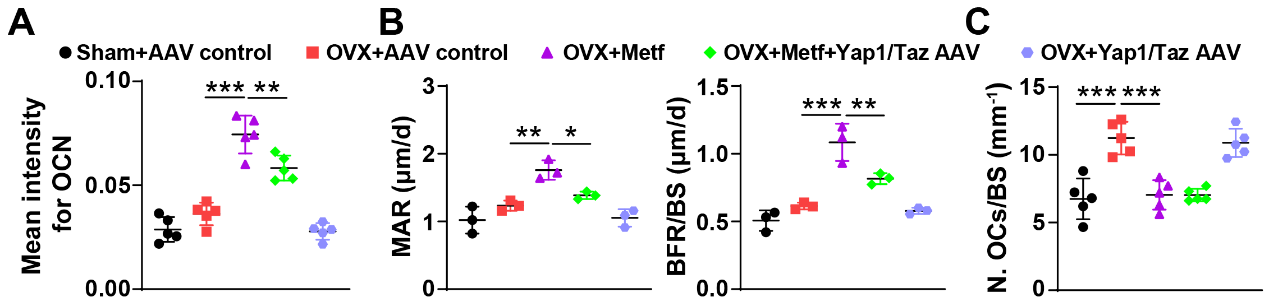
**Figure. S5 Overexpression of YAP1/TAZ hampers the osteogenic effect of metformin but does not affect osteoclastogenesis.**

**(A)** Quantitative analysis of the mean intensity of OCN in distal femora from different groups. n = 5 per group. **(B)** Mineral apposition rate (MAR) and bone formation rate per bone surface (BFR/BS) of trabecular bone measured by calcein double labeling. n = 3 per group. **(C)** Osteoclast number of trabecular bone surface measured by TRAP staining. n = 5 per group. Data are plotted as mean ± SD. ^*^*P* < 0.05, ^**^*P* < 0.01, ^***^*P* < 0.001.

**Table S1. Primer sequences for qRT-PCR.**

| **Gene** | **Forward (5’-3’)** | **Reverse (5’-3’)** |
| --- | --- | --- |
| *HIF1α* | AGAGGTTGAGGGACGGAGAT | GACGTTCAGAACTTATCCTACCAT |
| *YAP1* | GAACTGCTTCGGCAGGTGAG | GCAGGGCTAACTCCTGACATT |
| *WWTR1* | TCACATCCTGGCGACTCTCA | GAGGCCGGATTCATCTTCTGG |
| *CYR61* | TGGTCAAAGTTACCGGGCAG | GGCTCCATTCCAAAAACAGGG |
| *THBS1* | TTGGTCACCATGGGACATCTG | CAGGCACTTCTTTGCACTCATC |
| *VEGFA* | ACATCACCATGCAGATTATGCG | CTCCAGGGCATTAGACAGCA |
| *ANGPTL4* | AGACACAACTCAAGGCTCAG | CTCATGGTCTAGGTGCTTGTG |
| *GAPDH* | GGATTTGGTCGTATTGGGCG | TCCCGTTCTCAGCCATGTAGT |

**Supplementary table 2. siRNA target Sequences.**

| **siRNA** | **Target sequences** |
| --- | --- |
| si-*YAP1* #1 | CCACCAAGCTAGATAAAGA |
| si-*YAP1* #2 | GAGATGGAATGAACATAGA |
| si-*YAP1* #3 | GTAGCCAGTTACCAACACT |
| si-*WWTR1* #1 | CGATGAATCAGCCTCTGAA |
| si-*WWTR1* #2 | GGACAAACACCCATGAACA |
| si-*WWTR1* #3 | AGAGTCTGCTCTGAACAAA |
| si-*HIF1α* #1 | GGAACATGATGGTTCACTT |
| si-*HIF1α* #2 | CTACCCACATACATAAAGA |
| si-*HIF1α* #3 | CCAGCAACTTGAGGAAGTA |
